# The Role of the Oral Microbiota Related to Periodontal Diseases in Anxiety, Mood and Trauma- and Stress-Related Disorders

**DOI:** 10.3389/fpsyt.2021.814177

**Published:** 2022-01-27

**Authors:** María Martínez, Teodor T. Postolache, Borja García-Bueno, Juan C. Leza, Elena Figuero, Christopher A. Lowry, Stefanie Malan-Müller

**Affiliations:** ^1^Etiology and Therapy of Periodontal and Peri-Implant Diseases Research Group, University Complutense Madrid, Madrid, Spain; ^2^Department of Dental Clinical Specialties, Faculty of Dentistry, Universidad Complutense de Madrid, Madrid, Spain; ^3^Department of Psychiatry, University of Maryland School of Medicine, Baltimore, MD, United States; ^4^Military and Veteran Microbiome: Consortium for Research and Education, Aurora, CO, United States; ^5^Rocky Mountain Mental Illness Research Education and Clinical Center, Rocky Mountain Regional Veterans Affairs Medical Center, Aurora, CO, United States; ^6^Department of Pharmacology and Toxicology, Faculty of Medicine, Universidad Complutense de Madrid, Madrid, Spain; ^7^Hospital 12 de Octubre Research Institute (Imas12), Neurochemistry Research Institute, Universidad Complutense de Madrid, Madrid, Spain; ^8^Biomedical Network Research Center of Mental Health (CIBERSAM), Institute of Health Carlos III, Madrid, Spain; ^9^Department of Integrative Physiology, Center for Neuroscience, Center for Microbial Exploration, University of Colorado Boulder, Boulder, CO, United States; ^10^Department of Physical Medicine and Rehabilitation, University of Colorado Anschutz Medical Campus, Aurora, CO, United States; ^11^inVIVO Planetary Health of the Worldwide Universities Network, New York, NY, United States

**Keywords:** oral microbiota, anxiety disorders, trauma-related disorders, mood disorders, *Porphyromonas gingivalis*, *Aggregatibacter actinomycetemcomitans*, periodontitis

## Abstract

The prevalence of anxiety, mood and trauma- and stress-related disorders are on the rise; however, efforts to develop new and effective treatment strategies have had limited success. To identify novel therapeutic targets, a comprehensive understanding of the disease etiology is needed, especially in the context of the holobiont, i.e., the superorganism consisting of a human and its microbiotas. Much emphasis has been placed on the role of the gut microbiota in the development, exacerbation, and persistence of psychiatric disorders; however, data for the oral microbiota are limited. The oral cavity houses the second most diverse microbial community in the body, with over 700 bacterial species that colonize the soft and hard tissues. Periodontal diseases encompass a group of infectious and inflammatory diseases that affect the periodontium. Among them, periodontitis is defined as a chronic, multi-bacterial infection that elicits low-grade systemic inflammation *via* the release of pro-inflammatory cytokines, as well as local invasion and long-distance translocation of periodontal pathogens. Periodontitis can also induce or exacerbate other chronic systemic inflammatory diseases such as atherosclerosis and diabetes and can lead to adverse pregnancy outcomes. Recently, periodontal pathogens have been implicated in the etiology and pathophysiology of neuropsychiatric disorders (such as depression and schizophrenia), especially as dysregulation of the immune system also plays an integral role in the etiology and pathophysiology of these disorders. This review will discuss the role of the oral microbiota associated with periodontal diseases in anxiety, mood and trauma- and stress-related disorders. Epidemiological data of periodontal diseases in individuals with these disorders will be presented, followed by a discussion of the microbiological and immunological links between the oral microbiota and the central nervous system. Pre-clinical and clinical findings on the oral microbiota related to periodontal diseases in anxiety, mood and trauma- and stress-related phenotypes will be reviewed, followed by a discussion on the bi-directionality of the oral-brain axis. Lastly, we will focus on the oral microbiota associated with periodontal diseases as a target for future therapeutic interventions to alleviate symptoms of these debilitating psychiatric disorders.

## Introduction

Depression and anxiety disorders are among the most prevalent neuropsychiatric disorders (NPDs), with an estimated 322 million people living with depression and 264 million living with an anxiety disorder in 2015, according to the World Health Organization (WHO) global health estimates (https://apps.who.int/iris/bitstream/handle/10665/254610/WHO-MSD-MER-2017.2-eng.pdf) (1). Depressive disorders impose 1.84 disability-adjusted life years (DALYs) while anxiety disorders impose 1.13 DALYs as recently described (https://vizhub.healthdata.org/gbd-compare/) (2). These neuropsychiatric disorders are chronic and debilitating and many patients do not respond to or adhere to the available treatment options. Many patients suffering from depression experience relapse (3), with increased severity and resistance to treatment with each successive episode (4). Adherence to psychiatric medications is further hindered by their side-effect profiles (5), and, due to the limited efficacy of antidepressants, nearly a third of patients do not respond to treatment (6). Furthermore, the COVID-19 pandemic created an environment where several determinants of poor mental health are exacerbated resulting in a global increase of 27.6% in cases of major depressive disorder and a global increase of 25.6% in anxiety cases during the pandemic (7). The significant burden of these disorders and the current limitations in their treatment highlight the need to identify all role players in their etiology to discover novel therapeutic targets to lighten disease burden.

Beyond genetics, a common feature shared by many patients with NPDs is the early, intense or chronic exposure to physical and psychosocial stressors (8) and the underlying systemic inflammation and neuroinflammation (9, 10) caused by these exposures. However, the exact cause and mechanisms that drive this inflammatory profile are still under investigation. Growing evidence points to a potential role of the microbiota. One of the possible mechanisms whereby the microbiota could contribute to neuroinflammation is *via* circulating endotoxins, i.e., lipopolysaccharides (LPS), which are part of the outer membrane of Gram-negative bacteria, or other microbe- or pathogen-associated molecular patterns (MAMPs or PAMPs). In the case of LPS, toxicity is associated with the lipid component and immunogenicity is associated with the polysaccharide components, eliciting a variety of inflammatory responses (11). Endotoxins can enter the circulation more readily through compromised internal barriers, such as the oral and intestinal mucosa, thereby allowing toxins to spread systemically, resulting in an inflammatory cascade in the central nervous system (CNS). Indeed, leaky gut (due to compromised tight junctions in the intestinal lining) and subsequent bacterial translocation have been reported in depression and schizophrenia studies (12, 13). In this same line, the presence of a “leaky mouth” (due to the widening of the intercellular spaces between the epithelial cells and the rupture of the epithelium on the periodontal pocket in patients with periodontitis), has been proposed as being able to lead to the translocation of bacteria/inflammatory mediators across the oral mucosa into systemic circulation, and distant organs and tissues.

Research into the role of the gut microbiota in health and disease has skyrocketed in recent years, and more recently interest in the relationship between the oral microbiota and health and disease is on the rise. The description of the oral microbiota has changed in recent years with significant advances in methods that are used to study complex microbial ecosystems. The oral microbiota is organized in biofilms, defined as matrix-embedded microbial populations, adherent to each other and/or to surfaces or interfaces (14) (teeth, restorations, or soft tissues). A perturbation in these biofilms may lead to dysbiosis (where the equilibrium that usually exists between different bacteria is disturbed), which could influence the immune system to promote inflammation and ultimately drive the development of different diseases, such as dental caries, periodontal diseases, or peri-implant diseases.

There are different niches in the oral cavity due to the different environmental conditions or nutrient availability, for example, the subgingival or the supragingival niches, niches in dental fissures, tonsils or tongue, all bathed by saliva. Although while dealing with the study of oral microbiome it would be ideal to focus on each niche independently, there are also some publications considering the whole mouth as just one niche. In this review we are presenting both approaches depending on the information reported in the original studies, focusing on the subgingival niche whenever possible, as it is the one related to periodontal diseases. However, for those cases in which there was no direct evidence about the specific role of subgingival microbiota, the results on the oral niche as a whole are being reported.

Periodontal diseases encompass a group of infectious and inflammatory diseases that affect the tissues that surround and support the teeth, i.e., the periodontium, which is comprised of gingiva, cementum, periodontal ligament, and alveolar bone. During the 2017 World Workshop, jointly held by the American Academy of Periodontology and European Federation of Periodontology, a new classification of periodontal and peri-implant diseases and conditions was established. According to this new classification, two main types of periodontal diseases were identified: gingivitis and periodontitis (15).

Gingivitis is an inflammatory condition that remains contained within the gingiva and it is reversible. Clinically, it is manifested by redness and edema in the gingiva without clinical attachment loss. At a site level, dental biofilm-induced gingivitis is defined as “an inflammatory lesion resulting from interactions between dental biofilm, and the host's immune-inflammatory response, which remains within the gingiva and does not extend beyond to the periodontal attachment” (16). At a patient level, gingivitis is defined by the presence of at least 10% of bleeding locations, without clinical attachment loss (16).

On the other hand, periodontitis is characterized by loss of periodontal tissue support. Periodontitis is clinically demonstrated by an increased probing pocket depth (PPD), the presence of bleeding on probing (BOP) or radiographic alveolar bone loss (17). It is classified in different stages and grades. The stage (Stage I, II, III, or IV) classifies the severity and complexity of the disease, with Stage IV being the most severe and complex, consisting of advanced periodontitis with alterations of the masticatory function. Each stage is further defined as localized or generalized or having a molar-incisor pattern, depending on the number of teeth being affected. The grade (Grade A, B, or C) estimates the risk of disease progression and responsiveness to standard treatment, and the potential health impact of periodontitis, with grade C being the one with the most rapid rate of progression (18).

Even if the etiology of periodontitis is multifactorial, the presence of subgingival biofilm has been described as its primary etiological factor. In the context of periodontitis, biofilms may contain diverse bacterial taxa, but *Aggregatibacter actinomycetemcomitans* and *Porphyromonas gingivalis* have been considered keystone pathogens, which could drive oral dysbiosis by altering the host homeostasis, and subsequently resulting in disease. This chronic, multi-bacterial infection elicits low-grade systemic inflammation *via* the release of pro-inflammatory cytokines (19) and/or the direct invasion of periodontal pathogens in distant tissues (20, 21). The most studied inflammatory mediators include C-reactive protein and IL-6 (19, 22); however, the most recent evidence has introduced the importance of different inflammatory biomarkers in both serum and saliva of patients with periodontitis (nod-like receptor family pyrin domain-containing protein-3, transglutaminase 2 or galectin 3) (23–25).

Periodontitis has therefore been associated with both neurodegenerative (Alzheimer's disease) and NPDs (26, 27), as well as other systemic diseases like diabetes mellitus or cardiovascular disease (28–31), with periodontal pathogens being implicated in their etiology and pathophysiology by driving immune system dysregulation. Even if the association between periodontitis and neuropsychiatric diseases has been proven to be bidirectional, as patients with depression and stress may have a reduced self-care through amotivation (32), in this review we will be focused on one direction: the role that oral microbiota related to periodontal diseases has in anxiety, mood and trauma- and stress-related disorders due to the influence of this direction in the understanding of the relationship and its use for potential treatment.

Thus, this review aims to describe the known and potential molecular and cellular mechanistic components of the association between oral microbiota related to periodontal diseases and anxiety, mood and trauma- and stress-related disorders. The specific objectives of this narrative review are (a) to summarize the epidemiologic evidence about the association between periodontal diseases and anxiety, mood and trauma- and stress-related disorders; (b) to review the evidence from different model systems about the relationship between the dysbiosis of the oral microbiota related to periodontitis and the behavioral alterations characteristic of anxiety, mood and trauma- and stress-related disorders; (c) to relate the taxa implicated in periodontal health/ diseases as well as in anxiety, mood and trauma- and stress-related disorders; and (d) to analyze the potential role of the oral biofilm control in the therapeutics of anxiety, mood and trauma- and stress-related disorders.

## Epidemiology of Periodontal Diseases in Anxiety, Mood and Trauma- and Stress-Related Disorders

The study of the association between **periodontal diseases and psychosocial factors** is complex. Poor oral hygiene and periodontal status are prominent in individuals with NPDs (33). This poor oral health in individuals with NPDs is characterized by higher prevalence of dental caries and periodontal diseases. A meta-analysis by Kisely et al. reported greater dental decay and tooth loss in individuals diagnosed with anxiety and/or depression disorders (34). A case-control study in individuals with bipolar disorder reported an increased risk for periodontitis in the patient cohort compared to healthy controls. Furthermore, the depressive phase of bipolar disorder was significantly associated with periodontitis and total bacterial load (35). These findings correlate with other studies that reported poorer periodontal health and oral hygiene as well as higher prevalence of caries in bipolar patients compared to healthy controls (35). Several factors can contribute to this association between oral and mental health: poor nutrition and oral hygiene; high sugar consumption; comorbid substance misuse (such as tobacco, alcohol, or psychostimulants) and limited access to dental care (36–38). In addition, many neuropsychiatric patients suffer from dry mouth (xerostomia), a common side effect of psychotropic medications, with xerostomia being a major risk factor for oral health problems (39).

In 1993 Goldber created a bio-social classification model termed *common mental disorder*, which is characterized by a group of symptoms or features, such as displeasure, fatigue, insomnia, anxiety, or depression (40). Common mental disorder has also been associated with periodontitis in a cross-sectional study including 621 individuals from Brazil. In those patients with common mental disorder the crude probability of having periodontitis was 1.45 times higher than those without common mental disorder (41). Genco et al. (42) reported in a sample of 1,426 adult subjects, increased odds of severe periodontitis [Odds Ratio (OR) = 2.21; 95% Confidence Interval (CI): 1.11–4.38] among individuals with poor coping behaviors and under financial strain compared to subjects demonstrating good coping behaviors or without financial strain (42). Work-related or marital stress was also associated with a poorer periodontal condition (43, 44). Similar observations were made in individuals with war-related stress (45, 46). However, not every study was able to document that association. A systematic review of stress and psychological factors as possible risk factors for periodontitis was published in 2007 (47). More than 50% (57.1%) of the studies found a positive association, while 14.2% found a lack of association. No meta-analyses of observational studies are available about this specific association.

### Anxiety Disorders and Major Depression

Among all the neuropsychiatric diseases, major depression is the disorder that has been most extensively studied in its relation to periodontitis. There are three systematic reviews with meta-analysis focused on the epidemiological association between periodontitis and major depression. A summary of the methodology and results of these three systematic reviews is reported in Table 1.

**TABLE 1A:** Main characteristics of systematic reviews dealing with the association between periodontitis and depression: material and methods.

**Materials and methods**		
**References**	**Inclusion criteria**	**Exclusion criteria**
Araújo et al. (48)	(P) Patients: adult humans (E) Exposition: presence of depressive disorders (C) Comparison: control patients without depression (O) Outcomes: clinical periodontal parameters (PD, CAL, BOP) (S) Studies: cross-sectional, case-control, cohort studies; clinical trials	Case reports or reviews Other mental or systemic diseases Absence of healthy control patients Outcomes other than periodontitis
Liu et al. (49)	(P) Patients: adult humans (E) Exposition: chronic periodontitis (C) Comparison: periodontally healthy individuals (O) Outcomes: depressive and anxiety disorders measured by psychometric instruments (S) Studies: cross-sectional, case-control, cohort studies; clinical trials	Case reports, reviews, comments, or basic studies Other oral diseases Other neurological and psychotic disorders rather than emotional disorders Absence of healthy control patients
Zheng et al. (50)	(P) Patients: subjects aged ≥ 14 years (E) Exposition: depression and anxiety disorders (C) Comparison: individuals without mood and anxiety disorders (O) Outcomes: periodontal disease (S) Studies: cross-sectional, case-control, cohort studies	Case reports or comments, meeting abstracts, basic studies Other oral diseases Mood disorders other than depression or anxiety Absence of periodontal healthy controls Insufficient data Publication language other than English or Chinese

**TABLE 1B:** Main characteristics of systematic reviews dealing with the association between periodontitis and depression: results (risk of bias, diagnostic criteria).

**Results_included studies**			
**References**	Risk of bias assessment^*^	**Diagnostic criteria for periodontitis**	**Diagnostic criteria for depression**
Araújo et al. (48)	12/15 studies: low risk of bias 3/15 studies: high risk of bias	PD+CAL (4/7 studies) PD (7/7 studies) BOP (5/7 studies) Supragingival plaque (3/7 studies). Other: GI, PI gingival inflammation, subgingival calculus, number or present teeth… Less frequent: papillary bleeding index, approximal plaque index, bone loss in radiographs, vertical interproximal defects in panoramic radiographs, calculus, gingival recession, CPI, dental plaque, missing teeth, radiographic alveolar crestal height, gingival bleeding, periodontal index	10 different scales and inventories (geriatric depression scale, diagnostic and statistical manual for mental disorders, multidimensional coping inventory…)
Liu et al. (49)	14/14 studies: high quality (low risk of bias)	Chronic periodontitis (diagnosed by bleeding on probing, probing depth, clinical attachment loss, supragingival plaque, subgingival calculus, gingival bleeding, radiographic alveolar crest height, vertical bone defects >3 mm, number of standing teeth, GI, interradicular lesions etc.)	More than 20 different scales and inventories for anxiety and depression (brief symptom inventory, beck depression inventory, depression, anxiety and stress scale…) and self-reported depression
Zheng et al. (50)	Cross-sectional: 6/15 high risk of bias; 9/16 low risk of bias Case-control: 5/8 high risk of bias; 3/8 low risk of bias	CAL (>0, >2, ≥3, ≥4, ≥5), PPD (≥4, ≥5 ≥6), GI, PI, BOP, radiographic alveolar crestal height, CPI ≥3, panoramic radiography (>80% remaining bone), plaque control record, AAP, CDC, CI	Dichotomized. More than 20 different scales and inventories for anxiety and depression (brief symptom inventory, beck depression inventory, depression, anxiety and stress scale…) and self-reported depression

* *Newcastle–Ottawa scales with or without modifications*.

**TABLE 1C:** Main characteristics of systematic reviews dealing with the association between periodontitis and depression: results and conclusion remarks.

**Results_meta-analysis**					
**References**	**Cross-sectional studies**	**Case-control studies**	**Global**	**Amstar-2**	**Conclusion comment**
Araújo et al. (48)	*n* (s) = 7/*n* (*p*) = 6,125 OR = 1.03 (95% CI: 0.75–1.41); *I*^2^ = 60.34%	Meta-analysis not done due to high heterogeneity (87.48%) 3 studies reported positive significant association between depression and clinical parameters for periodontitis, while 3 studies showed no association.	/	High confidence	No association found when only cross-sectional studies are included in the meta-analysis
Liu et al. (49)	*n* (*s*) =7/*n* (*p*) = 5,997 OR = 1.03 (95% CI: 0.75–1.41)	*n* (*s*) = 6/*n* (*p*) = 994 OR = 3.72 (95% CI: 2.45–9.52)	*n* (*s*) = 14/*n* (*p*) = 7,139 OR = 1.61 (95% CI: 1.16–2.23) *I*^2^ = 84.3%, *p* < 0.001	Moderate confidence	When case-control studies are included in the analysis, a significant global association is found between periodontitis and depression
Zheng et al. (50)	*n* (*s*) = 17/*n* (*p*) = 23,838 OR = 1.08 (95% CI: 0.88–1.32); *I*^2^ = 71.8%, *p* < 0.001	*n* (*s*) = 8/*n* (*p*) = 1,245 OR = 1.70 (95% CI: 1.01–2.83); *I*^2^ = 72.5%, *p* = 0.001	*n* (*s*) = 25/*n* (*p*) = 25,083 OR = 1.16 (95% CI: 0.99–1.35) *I*^2^ = 70.9%, *p* < 0.001 Severe periodontitis subgroup: OR = 1.427 (95% CI: 1.053–1.933)	Moderate confidence	With a larger sample size, a significant association is found between periodontitis and depression, only when case-control studies are included in the analysis

*OR, odds ratio; CI, confidence interval; PD, probing depth; CAL, clinical attachment level; BOP, bleeding on probing; GI, gingival index; PI, plaque index; CI, calculus index; AAP, American Academy of periodontology; CDC, Centers for Disease Control and Prevention; s, studies; p, patients*.

**The inclusion criteria** of these studies were very similar, with slight differences related to age, study design or study outcomes. While Araújo et al. (48) and Liu et al. (49) included only adults, Zheng et al. (50) included patients ≥ 14 years old. All the reviews claimed in the inclusion criteria to select cross-sectional, case-control and cohort studies, and, although Araújo et al. (48) and Liu et al. (49) also looked for clinical trials, they finally included in the meta-analysis only cross-sectional and case-control studies. All included studies used healthy control patients as a comparison group, and depression was selected as exposure in Araújo et al. (48), while Zhen et al. (50) also included other mood disorders such as anxiety disorders. Liu et al. (49) presented periodontitis as exposure while the outcome was the affective and anxiety disorders (depression or anxiety).

By contrast, more important differences were found in terms of **diagnostic criteria** for periodontitis. The most commonly used clinical variables were PPD, BOP and clinical attachment level (CAL). Other common variables were gingival index (GI), supragingival dental plaque or radiographic alveolar crest height. In terms of the mental health diagnoses, most studies used more than one diagnostic questionnaire/scale. The studies included in Araújo et al. (48) used 10 different questionnaires for depression diagnosis whilst other meta-analyses included combined instruments. The studies included in Liu et al. (49) and Zhen et al. (50) not only used more than 20 different scales and inventories for anxiety and depression symptoms, but also self-reported depression.

In terms of **quantitative results**, Aráujo et al. (48) and Liu et al. (49) included the same 7 *cross-sectional studies* in their analyses and found a non-significant association between periodontitis and depression (OR = 1.03; 95% CI: 0.75–1.41). Similarly, Zheng et al. (50) also reported a non-significant association between periodontitis and depression (OR = 1.08, 95% CI: 0.88–1.32), despite the inclusion of additional studies and a bigger cohort in their analysis (17 studies, 23,838 participants). When *case-control studies* were included, both Liu et al. (49) and Zheng et al. (50) found a significant association between periodontitis and depression (OR = 3.72, 95% CI: 2.45–9.52 and OR = 1.70, 95% CI: 1.01–2.83, respectively). Araújo et al. (48) did not perform a meta-analysis of case-control studies due to high heterogeneity. One global meta-analysis by Liu et al. (49), including cross-sectional and case-control studies, found a significant association between periodontitis and depression (OR = 1.61, 95% CI: 1.16–2.23) (49), whereas, the global meta-analysis by Zheng et al. (50) reported a non-significant association between periodontitis and depression (OR = 1.16, 95% CI: 0.99–1.35). However, a subgroup analysis including only severe periodontitis revealed a significant association between these two conditions (OR = 1.43, 95% CI:1.05–1.93) (50).

In summary, the results of these systematic reviews and meta-analyses showed that data from case-control studies (which are more useful than cross-sectional studies in terms of hypothesis generation as they include a control group) show a positive association between depression and periodontitis (ORs = 1.17–1.70). Both the systematic reviews of Liu et al. (49) and Zheng et al. (50) performed meta-analysis regarding the association between anxiety (based on self-report questionnaire and/or clinician administered questionnaire scores) and periodontitis. The association between these diseases was more modest than the one with depression in the study by Liu et al. (49). For the cross-sectional studies (*n* = 2) they reported a non-significant association between anxiety and periodontitis (OR = 1.24, 95% CI: 0.63–2.43), and a significant association between anxiety and periodontitis (OR = 2.46, 95% CI: 1.17–5.13) for case-control studies (*n* = 5). In the case of Zheng et al. (50) this association could not be verified (OR = 0.70, 95% CI: 0.44–1.15) (*n* = 18).

Cohort studies were not included in these systematic reviews, as they are not very common. Hsu et al. (51) selected 12,708 patients with new periodontitis diagnosis and 50,832 matched periodontally healthy individuals. These patients were followed until the diagnosis of depression from 5 to 11 years. After the adjustment of sex, age and comorbidities, the group with periodontitis presented a 1.73-fold increased risk of developing depression [Hazard Ratio (HR) = 1.73, 95% CI: 1.58–1.89] (51). This finding strongly suggests that periodontitis could be a risk factor for the later development of depression. More cohort studies of this nature should be conducted for depression, and also for anxiety and trauma-related disorders.

### Post-traumatic Stress Disorder

The level of evidence for the association between PTSD and periodontitis is lower than that related to depression. It has only been studied in primary observational studies in adults and children, with different methodologies and contradictory results. These studies are summarized in Table 2.

**TABLE 2A:** Methodology and outcomes of publications studying the relationship between trauma-associated stress and periodontitis.

**Materials and methods**						
**References**	**Study desing**	**Country**	***N*** **(cases/** **controls)**	**Inclusion criteria**	**Exclusion criteria**	**Periodontitis definition**	**TAS definition**
Muhvić-Urek et al. (33)	Case-control	Croatia	100 (50/50)	**Cases**: men who experienced combat stress for more than 10 years and at the time of the study met the criteria for post-traumatic stress disorder **Controls**: men who did not participate in war	Not explicit	Periodontal characteristics of the groups (PI, CI, CPI)	Diagnosis of post-traumatic stress disorder from the international statistical classification of diseases and related health problems/structured clinical interview for diagnostic and statistical manual of mental disorders
de Oliveira Solis et al. (52)	Case-control	Brazil	76 (38/38)	**Cases:** patients with post-traumatic stress disorder diagnostic criteria **Controls**: healthy patients	Bipolar disorder, eating disorder, suicide risk, self-mutilating behaviors, lack of remembrance about the traumatic event, abuse or dependence of alcohol and drugs, patients with systemic disease that might have hindered periodontal clinical examination	Periodontal characteristics of the groups (CAL, PPD, BOP, Plaque)	Post-traumatic stress disorder module of the Structural Clinic Interview (DSM-IV-SCID) Davidson trauma scale (DTS)
Hamid and Dashash (53)	Case-control	Syria	60 (30/30)	Children that did not receive any treatment or medication **Cases**: diagnosed with post-traumatic stress disorder. **Controls**: healthy patients.	Other psychiatric disorders	PI and GI at 4 points/tooth. Patients were classified in mild (0–1), moderate (1.1–2) and severe (2.1–3) plaque and inflammation	Full criteria for post-traumatic stress disorder by 2 independent psychologists (5th edition of diagnostic and statistical manual of mental disorders). Child post-traumatic stress reaction index questionnaire to assess severity.
Tagger-Green et al. (54)	Cross-sectional	Israel	71	Combat related post-traumatic stress disorder patients for at least 10 years	Not explicit	American Academy of Periodontology (55). Localized (<30%) or generalized (>30%). Slight (1–2 mm CAL), moderate (3–4 mm CAL), severe (>5 mm CAL)	Full criteria for post-traumatic stress disorder according to the Diagnostic and Statistical Manual of Mental Disorders, 4th edition (DSM-4)

**TABLE 2B:** Methodology and outcomes of publications studying the relationship between trauma-associated stress and periodontitis.

**Results**				
**References**	**Quantitative results**	**Qualitative results**	**Nos**.	**Conclusion comment**
Muhvić-Urek et al. (33)	/	Statistically significant differences in PI and GI between cases and controls. Statistically significant differences in CPI between cases and controls. The majority of cases were classified with CPI value 3 (36%), while the controls were classified in majority with CPI 2 (46%)	4	The periodontal outcomes among cases are poorer than among controls, with higher plaque and gingival index, and CPI values
de Oliveira Solis et al. (52)	CAL: cases-2.46 (0.98)/controls-2.19 (0.58) *p* = 0.055 PPD: cases-2.07 (0.42)/controls-2.01 (0.41) *p* = 0.623 BOP: cases-15.89 (13.11)/controls-14.47 (9.57) *p* = 0.937	Prevalence CAL (%subjects) ≥ 4: cases-85.71%/controls- 78.95% *p* = 0.450 Prevalence CAL (%subjects) ≥ 5: cases-57.14%/controls- 44.74% *p* = 0.290 Prevalence CAL (%subjects) ≥ 6: cases-37.14%/controls- 28.95% *p* = 0.456 Prevalence PPD (%subjects) ≥ 4: cases-65.71%/controls- 73.68% *p* = 0.458 Prevalence PPD (%subjects) ≥ 5: cases-34.29%/controls- 34.21% *p* = 0.995 Prevalence PPD (%subjects) ≥ 6: cases-17.14%/controls- 21.05% *p* = 0.672	4	There are no differences related to periodontal clinical parameters between cases and controls
Hamid and Dashash (53)	/	Statistically significant differences in PI and GI between cases and controls. Severe PI occurred in 16.7% of cases and 0% of controls (*p* < 0.001) Severe GI occurred in 33.4% of cases and 6.7% of controls (*p* < 0.001)	6	PTSD children had a poorer gingival status than matched controls and they were affected by PTSD severity
Tagger-Green et al. (54)	/	All the patients had periodontitis. 70.4% had localized periodontitis, and 29.6% had generalized form. 66.2% of patients had severe disease; 25.4% had moderate disease; and 8.5% had mild disease. 70.4% had a plaque index > 0.8	3	High rate of severe periodontitis among post-traumatic stress disorder patients, even if most patients had localized periodontitis.

*TAS, trauma-associated stress; PI, plaque index; CI, calculus index; CPI, community periodontal index; GI, gingival index; CAL, clinical attachment level; PPD, probing pocket depth; BOP, bleeding on probing; NOS, Newcastle-Ottawa Scale*.

A case-control study conducted in a Brazilian cohort reported no significant differences in CAL, PPD or BOP between healthy controls (*n* = 39) and patients with PTSD (*n* = 38) (52). With a larger sample (*n* = 50 patients with PTSD and *n* = 50 healthy controls), Muhvić-Urek et al. (33) found statistically significant differences between PTSD patients and controls in plaque index (PI), GI and community periodontal index (CPI). Most of the PTSD patients were classified with CPI 3 (36%), which implies the presence of periodontal pockets of 4–5 mm and consequently, the presence of periodontitis, while controls were mostly classified with CPI 2, characterized by the presence of shallow pockets although calculus and inflammation are present. Statistically significant differences have been confirmed in a case-control study in Syria, with children diagnosed with PTSD compared to healthy children. All of them were classified into mild, moderate, or severe dental plaque and gingival inflammation. Severe periodontal indices occurred more frequently in patients diagnosed with PTSD (16.7% of cases and 0% of controls for severe PI; and 33.4% of cases vs. 6.7% of controls for severe GI) (53). A cross-sectional study in Israel also concluded that a high rate of severe periodontitis was evident among 71 patients with a diagnosis of combat-related PTSD. The overall prevalence of periodontitis, according to the criteria established by the American Academy of Periodontology in 1999 (55), was 100% among these patients, with 66.2% of patients having severe periodontitis (54). This is a high prevalence of periodontitis when compared with the one in the general population, with an estimated prevalence of 29.4% for periodontitis and a 9.8% for severe periodontitis (56).

In summary, these results suggest an association between periodontal diseases and PTSD, however, the current studies are highly heterogeneous and were conducted in relatively small cohorts. Future studies should include longitudinal designs with meticulous methodology in large cohorts to clarify the association between periodontal diseases and PTSD and disentangle causes from consequences.

## Immunological Links Between Periodontitis and Anxiety, Mood and Trauma- and Stress-Related Disorders

**Figure 1:**
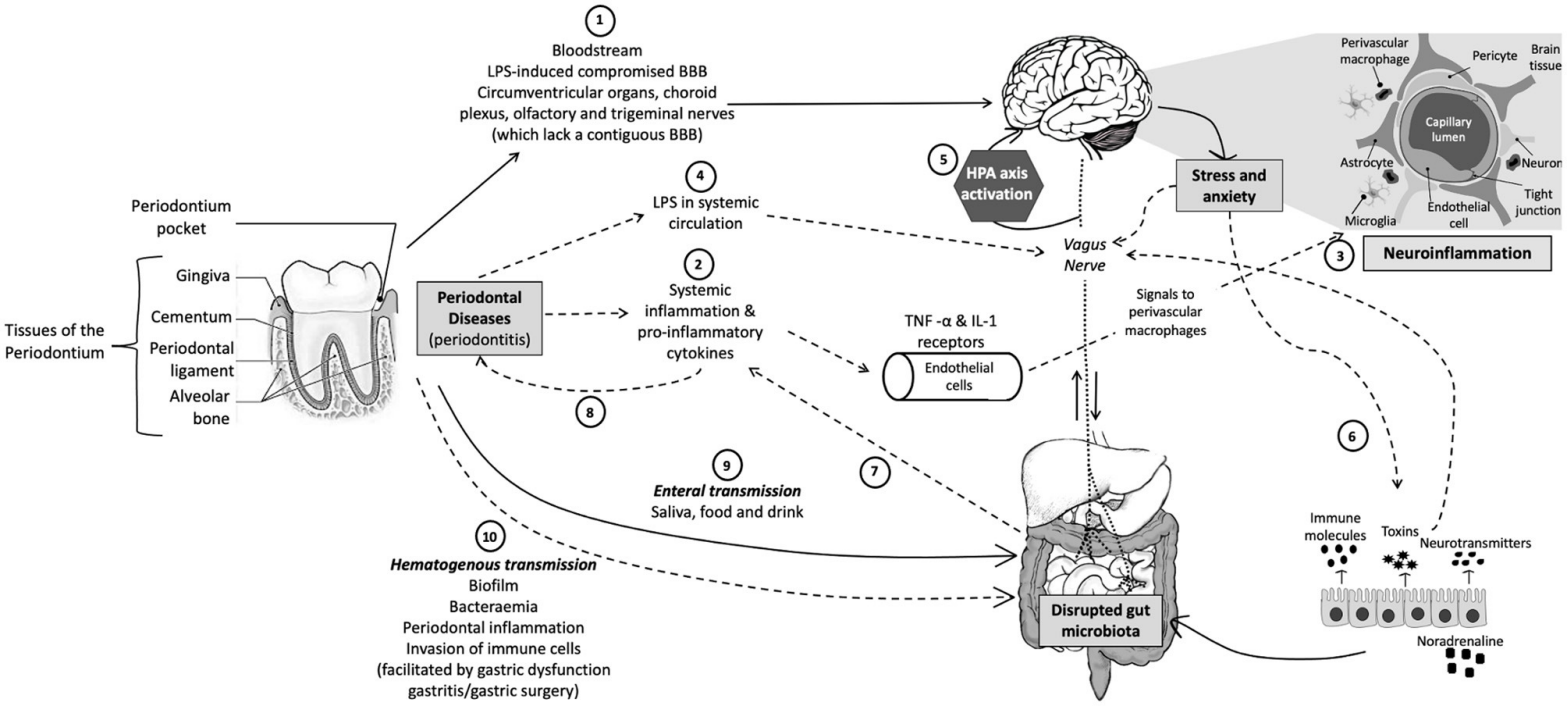
Schematic representation of the oral-gut-brain axis. (1) Periodontal bacteria can directly reach the brain *via* the bloodstream or areas that lack an intact BBB or with a compromised BBB. (2) Periodontitis can indirectly affect the CNS *via* pro-inflammatory cytokines that activate endothelial cells that express TNF-α and IL-1 receptors, which, in turn, signal to the perivascular macrophages that communicate and activate microglia, resulting in (3) neuroinflammation. (4) Periodontitis can also result in leaky periodontium and LPS in systemic circulation, which can (5) activate the HPA axis and result in increased stress hormones or neurotransmitters, which subsequently (6) influence gut physiology, microbiota habitat, microbiota community composition, and bacterial gene expression. (7) An altered gut microbiota can result in systemic inflammation, which not only affects the CNS but also (8) exacerbates other inflammatory pathologies, such as periodontitis. Periodontal bacteria can directly influence gut microbial community composition and functioning *via* (9) enteral transmission or indirectly *via* (10) hematogenous transmission (which is facilitated by conditions such as gastritis, gastric surgery, or gastric dysfunction). CNS, central nervous system; TNF, tumor necrosis factor; IL-1, interleukin-1; LPS, lipopolysaccharide; BBB, blood–brain barrier; HPA, hypothalamic–pituitary–adrenal. Solid arrows indicate direct pathways and dotted arrows indicate indirect pathways.

Although few studies have evaluated the relationships among the oral microbiota in periodontal diseases, and anxiety disorders, mood disorders, and trauma-related disorders, such as PTSD (see section Clinical Investigations of Oral Microbiota Composition in Mood, Anxiety, and Trauma-Related Disorders below), there is sound theoretical basis to hypothesize relationships among these conditions and providing a rationale for future studies. One hypothetical framework that supports these relationships is emerging evidence that poor oral health and periodontitis can lead to systemic chronic low-grade inflammation (57), which is an important risk factor for NPDs (58, 59). Chronic psychosocial stress may be a common risk factor for both conditions (8, 60) (Figure 1).

Systemic chronic low-grade inflammation is now increasingly recognized as a risk factor for anxiety disorders (61), mood disorders (62–66), and trauma-related disorders (67). A transdiagnostic meta-analysis (i.e., a meta-analysis conducted across diagnostic criteria for multiple stress-related psychiatric disorders) of the relationship between biological signatures of inflammation and stress-related psychiatric disorders revealed that, across *k* = 36 independent samples and *n* = 14,991 participants, trauma exposure was positively associated with plasma or serum concentrations of C-reactive protein (CRP), interleukin (IL)-1β, IL-6, and tumor necrosis factor (TNF)-α (68). These studies however may only be part of the story as, while individuals with a diagnosis of major depressive disorder, for example, have elevated baseline plasma concentrations of IL-6, they have brisker and ampler IL-6 increases in response to exposure to psychosocial stress (i.e., in the Trier Social Stress Test paradigm). It may be that the exaggerated psychosocial stress-induced inflammation is more important than chronic low-grade inflammation itself but determining this will require more studies evaluating the effects of acute psychosocial stress on biological signatures of inflammation in individuals with stress-related psychiatric disorders. Nevertheless, baseline biomarkers of inflammation, including CRP and IL-6, can predict future depressive symptoms, up to a decade later, in both children (69) and adults (70).

Reinforcing the hypothesis that inflammation is an important risk factor for development of stress-related psychiatric disorders, a recent study using machine learning approaches determined that biological signatures of inflammation prior to deployment were among the highest-ranking features predicting development of PTSD symptoms following deployment in active military personnel (71). In section Clinical Investigations of Oral Microbiota Composition in Mood, Anxiety, and Trauma-Related Disorders, below, we will evaluate the evidence that periodontitis, and associated changes in the oral microbiota, may be one important source of chronic low-grade inflammation contributing to elevated risk of developing anxiety disorders, mood disorders, and trauma-related disorders, such as PTSD.

## Models That Study the Oral Microbiota and Periodontitis in the Context of Behavioral Outcomes Related to Anxiety, Mood and Trauma- and Stress-Related Disorders

In order to assess the role of the oral microbiota in anxiety, mood and trauma- and stress-related disorders, different pre-clinical models have been used. There are multiple pre-clinical models for the induction of periodontal diseases, using different species and methodologies, each with its inherent limitations. Some models are more suitable for the evaluation of behavioral outcomes in combined models of periodontitis and anxiety or mood and trauma-related disorders, due to their similarities to the human disease. In this group we can find studies in non-human primates, dogs and pigs. For example, the periodontitis model in beagle dogs has been extensively used as they naturally develop periodontitis. However, these models are not ideal for studies on periodontal medicine due to ethical considerations as well as financial and time constraints (72).

At the opposite end we can find the *in vitro* models in which cultures of different cell types are exposed to single species of bacteria classically related to periodontal diseases such as *P. gingivalis* or *Fusobacterium nucleatum* (*F. nucleatum*); or in combination (trying to resemble the situation of the biofilm in periodontitis patients). These *in vitro* models are typically used to evaluate and identify putative molecular/cellular mechanisms that could be explored in relation to behavioral alterations in subsequent *in vivo* models (73). Another interesting approach is the development of *in vitro* periodontal biofilm models to test the antimicrobial/anti-inflammatory profile of different compounds and therapeutic strategies (74–76).

At a mid-point are *in vivo* models using rodents. Rodents are often used to evaluate behavioral outcomes; these models retain translational potential and are less demanding on resources (77). Therefore, these models are the most often used to concomitantly explore molecular and cellular mechanisms related to disease phenotypes and behavioral alterations. The main challenges in these models are the minimal amount of gingival tissue available and that rodents are endogenously resistant to the development of periodontal diseases. In some cases the methodologies to cause disease are difficult to manage, and different protocols have emerged to induce periodontal diseases (78): (1) silk 3.0, nylon 3.0 or cotton 3.0 ligatures of a maxillary molar tooth; (2) oral gavages during several weeks with bacteria such as *P. gingivalis, A. actinomycetemcomitans*, or *F. nucleatum* or a mixture of these taxa; (3) combined methodology of impregnating ligatures with a bacteria enriched solution; (4) injection of LPS into the gingival tissues or into the connective tissue overlying calvarial bone; and (5) surgical periapical dental lesion.

With these different models, the relationship between the dysbiosis of the oral microbiota and the behavioral alterations characteristic of anxiety, mood and trauma- and stress-related disorders have been analyzed. Only some studies have directly evaluated this relationship. Most of them assessed mechanisms that have the potential to be involved in behavioral alterations [e.g., alteration in the immune and hypothalamic-pituitary-adrenal (HPA) stress axis responses]. Below we focus on findings from studies using non-human primates and rodents.

### Findings in Non-human Primates

Pre-clinical studies on periodontal diseases in non-human primates mostly use a ligature-induced periodontitis protocol in *Macaca mulatta*. To the best of our knowledge there is not documented subsequent development of neuropsychiatric symptoms in these animals with periodontitis; however, recent data have shown that this ligature model induces an increase in follicular helper T cell (Tfh) responsiveness during the progression of periodontitis, which was found to be affected by age and the specific microbial complexes present in the oral microbiome (79). Follicular helper T cells are a specialized subset of CD4+ T cells that play a critical role in protective immunity helping B cells produce antibodies against foreign pathogens. Recently, it has been shown that a particular subset of Tfh cells infiltrate the brain of mice suffering systemic lupus erythematosus, thereby possibly contributing to the neuropsychiatric manifestations (memory impairment, cognitive deficits and mood disturbance) of this disease (80). Some of these symptoms are also present in anxiety, mood and trauma and stress-related disorders (81).

### Findings in Rodents

Recently, anxiety-like behaviors [assessed using the elevated plus-maze (EPM)] have been reported in Wistar rats submitted to a ligature-induced pre-clinical model of periodontitis in the acute phase (14 days), but not in the chronic phase (28 days) of the disease (82), stressing the importance of timing in this type of longitudinal study to assess anxiety-like defensive behavioral responses, and other behaviors relevant to psychiatric disorders, in an animal model of periodontitis.

Similar depressive- and anxiety-like behaviors have been described in rats subjected to experimental periapical lesions; these behaviors were effectively reverted by the administration of the antidepressant imipramine, possibly via stress modulation and, in part, via immune modulatory mechanisms (83).

Models using pathogenic periodontal bacteria are the ideal when studying the possible role of oral microbiota on systemic health. Even if the use of ligatures to induce periodontitis has demonstrated more severe periodontal destruction in shorter time, oral gavages with periodontal pathogens or their endotoxins (such as LPS) are more representative of the physiopathology of periodontitis (84). LPS-induced periodontitis may promote the occurrence and progression of learning and memory impairment by modulating the T helper 17 (Th17)/regulatory T cell (Treg) balance in a mechanism related to the STAT3 signaling pathway (81). In a similar way, C57BL/6 mice injected with *P. gingivalis* exhibited impaired spatial learning and memory during the Morris Water Maze test and exhibited attenuated passive avoidance learning, effects that were mediated by the activation of the toll-like receptor (TLR) 4 pro-inflammatory signaling pathway (85). It is worth mentioning that the animals were also submitted to the open-field test (OFT), and no significant differences were observed between control and LPS groups, indicating that the anxiety-like behavior and the activity of the mice was not affected by purified *P. gingivalis*-LPS. Another study reported the induction of depressive-like behavior (measured by tail suspension test and forced-swim test) in *P. gingivalis*-treated mice and related these behavioral changes to an increased number of activated astrocytes and decreased levels of mature brain-derived neurotrophic factor (BDNF) and astrocytic p75 neurotrophin receptor in the hippocampus (86). Again, OFT and EPM were performed to evaluate anxiety-related behaviors and there were no differences between *P. gingivalis*-treated and control mice. These last studies suggest the need to differentiate depressive-like symptoms from specific anxiety-like behavioral alterations, seeing that their results showed that purified *P. gingivalis*-LPS-induced periodontitis induced strong features of depression, but to a lesser extent features of anxiety (85, 86).

Very few studies have been conducted using combined animal models of stress exposure as a trigger of anxiety-related behaviors together with periodontitis induction; these designs are fundamental to disentangle the bi-directional relationship between these pathological conditions. Some authors have reported that the psychotropic drugs diazepam and fluoxetine, commonly prescribed in the treatment of anxiety, mood and trauma- and stress-related disorders, induce an anti-inflammatory response and reverse the alveolar bone loss that is enhanced by conditioned fear stress in a ligature-induced periodontal disease model in rats (87, 88). Following this approach, conditioned fear stress with electrical shocks prior to a periapical lesion increased the size of bone loss in Wistar rats, with a concomitant increase of inflammatory cells in the affected tissue (89). On the contrary, a well-known anxiolytic activity, like mild physical training, attenuates alveolar bone loss and anxiety-like behavior in rats with periodontitis induced by a ligation protocol in both first mandibular molars (90). These beneficial actions could be related to the ability of mild exercise to increase the levels of the anti-inflammatory cytokine IL-10 and therefore likely Treg function, which has also been considered for a long time as a therapeutic target for future interventions in stress-related neuropathologies (91).

Exposure to chronic emotional stress affects the humoral immune response to *P. gingivalis* in mice, shifting the response toward a Th1-type response (92). Alterations in the Th1/Th2 response balance have been found in several NPDs, including anxiety, mood and trauma- and stress-related disorders (61).

Other authors have used different stress-based models that are aimed at inducing endophenotypes relevant to depression, such as exposure to 21 consecutive days of Chronic Mild Stress (CMS) or olfactory bulbectomy. Martinez et al. (93) induced periodontitis in rats using oral gavages with *P. gingivalis* and *F. nucleatum* over 12 weeks, followed by a 3-week period of CMS induction. They reported that the animals submitted to both pathological stimuli presented the highest expression of pro-inflammatory mediators [TNF-α, IL-1β and nuclear factor kappa-light-chain-enhancer of activated B cells (NF-kB)] in the frontal cortex, dysregulation of the HPA stress axis, presence of *F. nucleatum* in the brain parenchyma and increased periodontal clinical variables such as alveolar bone levels, when compared with the control group without periodontitis or CMS. Interestingly, the presence or absence of periodontitis did not modify anxiety- and anhedonia-like behaviors and weight loss produced by CMS exposure (93).

Using the olfactory bulbectomy model of depression, the authors found that animals submitted to depression and periodontitis showed increased alveolar bone loss, depressive-like behaviors, HPA axis dysregulation and susceptibility to an inflammatory stimulus such as LPS. In addition, they found that the treatment with an antidepressant, tianeptine, prevented all these negative effects, except the dysregulation of the HPA axis (94). The same authors also performed neonatal exposure to LPS from *Salmonella enteritidis*, as a stress-related stimulus (immunological stress and HPA axis activator) prior to silk ligature-induced periodontitis (95). LPS pre-treated animals showed increased periodontal attachment loss and higher plasma IL-6 levels. Furthermore, significant correlations were noted between specific emotional-/anxiety-related behaviors and the severity of periodontal disease in terms of attachment loss.

Finally, some authors have used an *in vivo/ex vivo* methodology: first they expose mice to a social stressor, followed by evaluation of anxiety-like behaviors and sacrifice, during which spleens were harvested and spleen cells were stimulated *ex vivo* with LPS derived from *P. gingivalis*. This approach allowed the authors to discover a higher pro-inflammatory profile in the CD11b+ spleen cells of stress-exposed mice that received LPS treatment (96). Interestingly, the presence of peripheral myeloid (CD11b^+^) cells in the brain has been related to anxiety-like behaviors in mice exposed to social stress (97).

In summary, the models in rodents have been the most widely used in the study of the relationship between periodontitis and depression and anxiety related disorders. The combined models have shown HPA axis dysregulation together with an hyperinflammatory response that sometimes was accompanied by behavioral changes. More studies are needed in the future to determine the precise nature of the bidirectional relationship between periodontitis and stress-related neuropsychiatric pathologies, to explore the possible mechanism/s involved in the transport and signaling of the oral microbiota into the CNS and, finally, to explore new therapeutic agents for the individual or combined treatment of these pathologies.

## Clinical Investigations of Oral Microbiota Composition in Mood, Anxiety and Trauma-Related Disorders

Although at first glance it might seem that poor mental health is the driver in the relationship between oral and mental health, a large-scale (*n* > 60 000), longitudinal (10-year follow-up) study detected that there was a higher incidence of *subsequent* development of depression in individuals in the periodontitis group compared to the non-periodontitis group (as discussed in detail earlier). This finding provides evidence that periodontitis could be a risk factor for the later development of major depression (51). It is therefore possible that periodontitis may be a susceptibility factor in mental health conditions. The question therefore arises: what are the mechanisms involved whereby poor oral health and periodontal diseases can impact CNS functioning and mental health?

The human oral bacterial community might hold some clues; great advances have been made to comprehensively catalog the diversity of bacteria and archaea in the human mouth and to identify associations between specific taxa and health and disease states (98, 99) (Table 3). However, limited data are available for the diversity and community composition of the oral microbiota in individuals with NPDs. A study that investigated the oropharyngeal microbiota of individuals with schizophrenia (*n* = 121), mania (*n* = 62), major depressive disorder (*n* = 48) and controls (*n* = 85) found that the microbiota community composition and relative abundances of specific taxa differed between the schizophrenia and mania cohorts and controls and that five taxa differed among the diagnostic groups. In the schizophrenia and mania cohorts *Prevotella, Neisseria subflava* and *Weeksellaceae* were decreased compared to controls, whilst *Streptococci* was increased compared to the control group. *Schlegelella*, was only present in individuals with mania and *N. subflava* was positively associated with cognitive functioning. The authors did not detect any significant differences in relative abundances of specific taxa between individuals with major depression and controls (103). The lack of significant findings in the depression cohort could be due to the small sample size of this cohort.

**TABLE 3:** Summary of taxa implicated in periodontal diagnoses as well as anxiety disorders, depressive disorders, and trauma- and stressor-related disorders.

**Taxa**	**Results**	**References**
*Prevotella*	*Prevotella intermedia*, and *Prevotella nigrescens* was correlated with diseased periodontal tissues	(100, 101)
	Negatively associated with distress and negatively associated with inflammatory markers and part of a consortium of taxa that could accurately predict distress and inflammation (saliva samples)	(102)
	Decreased in schizophrenia and mania cohorts (oropharyngeal samples)	(103)
*Neisseria*	Decreased in schizophrenia and mania cohorts (oropharyngeal samples)	(103)
	*Neisseriales* order was positively associated with CRP; *Neisseriaceae* family was positively associated with CRP and cortisol (saliva samples)	(104)
*Fusobacterium*	*Fusobacterium nucleatum* was correlated with diseased periodontal tissues	(105)
	Positively correlated with depression (saliva samples)	(104)
*Leptotrichia*	Associated with persistent generalized periodontal disease	(105)
	Positively correlated with depression and cortisol levels (saliva samples)	(104)
	Positively associated with distress and host inflammation (saliva samples)	(102)
*Streptococcus*	Higher proportions in patients successfully treated using active periodontal treatment (saliva samples)	(105)
	A *Streptococcus* taxon was associated with cortisol, anxiety and depression	(104)
	*Streptococcus* taxa could accurately predict distress	(102)
*Selenomonas*	Associated with persistent generalized periodontal disease	(105)
	Implicated to be involved in the pathogenesis of periodontitis	(106–108)
	A *Streptococcus* taxon was associated with cortisol, anxiety and depression	(104)

*CRP, C-reactive protein*.

Another study that focused on self-reported anxiety and depressive symptoms in an adolescent cohort found no difference in the diversity of the oral microbiome (using saliva samples) in the anxiety or depression groups compared to controls (104). However, depression and anxiety symptoms were associated with differential relative abundances of particular taxa. The relative abundance of *Spirochaetaceae* was positively correlated with both anxiety and depression and the relative abundances of *Actinomyces, Fusobacterium* and *Leptotrichia* spp. were positively correlated with depression. A positive correlation between the relative abundance of *Leptotrichia* spp. and cortisol levels was also noted. The relative abundances of Actinobacteria and Gracilibacteria phyla were positively correlated with basal cortisol levels, and the relative abundance of Proteobacteria positively correlated with basal CRP, a marker of inflammation. The relative abundance of Actinomycetales order was positively associated with cortisol, and the relative abundance of Neisseriales order was positively associated with CRP. The relative abundance of *Neisseriaceae* family was positively associated with CRP and cortisol. The relative abundances of a total of 16 species were significantly associated with cortisol, including some that were associated with anxiety and depression symptoms, such as *Lachnoanaerobaculum orale*, a *Streptococcus* taxon, and a *Selenomonas* taxon. No significant associations between self-reported oral health and relative taxonomic abundances of specific taxa were observed (104). The authors concluded that oral microbial composition was associated with adolescent anxiety and depression symptoms. Since this was a cross sectional study, the driving factors for associations between bacterial taxa and stress and inflammatory markers cannot be delineated, underscoring the need for longitudinal study designs. It is imperative to build upon this research, especially since adolescence is an essential developmental period in which to identify early targets for intervention (109).

Kohn et al. investigated the associations between distress, the oral microbiota and inflammatory markers in healthy adults, with saliva samples collected at five time points within 1 day. The authors reported a diurnal pattern in the relative abundances of specific taxa over time: *Neisseria, Prevotella*, and *Bacertoides* were strongly associated with waking samples and *Veillonellaceae, Ruminococcaceae*, and *Sphingomonas* were associated with later timepoints. Individuals with high distress exhibited greater alpha diversity compared to individuals with low distress; furthermore, high distress individuals had less diurnal variance in their community structure compared to low distress individuals. The authors commented that it is possible that lower alpha diversity in saliva could be associated with better oral health (110), which is contradictory to what has been observed in the gut microbiota (111), and therefore requires further investigation. Additionally, individuals with high levels of distress also displayed greater homogeneity in microbial community structure. The relative abundances of *Leptotrichia, Bacteroidetes, Selenomonas*, and *Haemophilus* were positively associated with distress, while the relative abundance of *Prevotella* was negatively associated with distress. The relative abundances of *Leptotrichia, Capnocytophaga, Treponema*, and *Bacteroidetes* were positively associated with host inflammation, whereas the relative abundances of *Aggregatibacter, Bifidobacterium, Prevotella*, and *Veillonella* were negatively associated with inflammatory markers. About 22% of taxa were significantly associated with both distress and inflammation and included features from Bacteroidetes and *Leptotrichia* taxa (102). Three features from the *Sphingomonadaceae* family as well as taxa from *Prevotella, Neisseria, Streptococcus*, and *Caulobacter* genera could accurately predict distress. Two features of the *Sphingomonadaceae* family and the *Staphylococcus, Prevotella* and *Caulobacter* genera could accurately predict the level of inflammation. Three features were shared between the distress and inflammation classifiers: one from the *Caulobacter* genus and two from the *Sphingobium* genus. The authors also reported that distress scores were associated with increased steroid and tryptophan degradation and reduced tryptophan biosynthesis. Host inflammation was associated with lower steroid and tryptophan degradation and increased LPS biosynthesis. Of the top 12 pathways shared between distress and inflammation, all had opposite relationships with each factor (e.g., positive association with inflammation but negative association with distress) (102), which is interesting given that, as noted above, several studies have reported a strong association between stress-related disorders and a pro-inflammatory state (61, 62, 65–67, 112, 113).

It is however important to note that most studies used saliva samples or supragingival samples when investigating the oral microbiota. However, the ideal area to focus on in the context of periodontitis is the subgingival microbiota. One study used subgingival samples to investigate whether there were associations between psychosocial factors scores or salivary cortisol levels and clinical periodontal parameters and microbiota in periodontitis patients (114). Subgingival microbiota samples were collected in two pathological sites (PPD ≥ 5 mm) and one healthy site of diseased patients (*n* = 30) [before/after scaling and root planing (SRP)] and from one healthy site from control patients (*n* = 30) (samples collected before/after SRP). Although they did not detect a correlation between salivary cortisol and self-reported stress/anxiety, cortisol levels were positively associated with periodontal pocket depth. Furthermore, high levels of *Tannerella forsythia* were present in the periodontal pocket samples of all highly stressed patients compared to those with low stress levels and *A. actinomycetemcomitans* was only detected in the pockets of non-anxious patients (114).

These findings provide insight into the possible mechanisms whereby the oral microbiota could influence CNS functioning and mental health outcomes, and future functional studies should build on these reported associations to determine how these oral taxa influence inflammation, and levels of LPS and tryptophan. An important aspect that should be considered in oral microbiota studies is that the oral cavity consists of multiple unique environments, each with environmental and nutritional conditions that allow the colonization of particular microorganisms. Therefore, each niche: gingival sulcus, periodontal pocket, cheeks, tongue, hard and soft palate, throat, surface of the teeth and saliva, should be sampled with the appropriate equipment to be able to accurately study each niche area. Since these microhabitats harbor different microbial communities (98), studies should ideally analyze them separately using appropriate sampling techniques, for example, sterile Gracey curettes to collect biofilm on teeth and hard tissues, sterile brushes for oral mucosa, or sterile paper points for gingival crevicular fluid (GCF) samples (115, 116).

## Bi-Directionality of the Oral-Gut-Brain Axis

Pre-clinical and clinical research have clearly illustrated the importance of the gut microbiota in CNS functioning and behavior; communication between the gut and the CNS is primarily mediated through neural, immune, and neuroendocrine pathways within the bidirectional microbiota-gut-brain axis (117–121). However, little is known regarding the role of the periodontal microbiota in mental health; the connection between the oral and gut microbiotas; its impact on CNS functioning and, in turn, how stress and anxiety impact the oral microbiota. Refer to Figure 1 for a visual illustration of the following sections.

### The Oral-Gut Connection

In order for oral microbes to reach and colonize the gut, they have to travel *via* the acidic and nitrite-rich environment of the stomach and the majority of microorganisms do not survive this environment. Although it was a long-held conception that the stomach is a relatively sterile organ due to its acid production, the human stomach also has a core microbiome, dominated by five major phyla: Firmicutes, Bacteroidetes, Actinobacteria, Fusobacteria, and Proteobacteria (122). Two possible routes of translocation of bacteria from the oral cavity to the gut have been proposed: hematogenous (*via* the bloodstream) and enteral (*via* the gastrointestinal tract) routes.

#### Hematogenous Route

In patients with periodontitis with a dislaceration of the epithelium of the periodontal pockets, daily dental hygiene practices (hard brushing/flossing) or invasive dental procedures could enable oral bacteria to spread into systemic circulation (bacteremia) (123, 124). Studies have shown that periodontal inflammation triggers the spread of oral bacterial to the liver and spleen (125). Furthermore, oral bacteria have the ability to invade and survive inside immune cells, such as macrophages and dendritic cells, thereby enabling oral bacteria to use host immune cells to serve as Trojan horses for transmission from the oral to the gut mucosa (126).

#### Enteral Route

Daily, humans swallow about 1.5 liters of saliva that is rich in oral bacteria (127, 128). However, due to the barrier functions along the gastrointestinal tract and acidity of the stomach, ingested oral bacteria seldom reach and colonize the healthy gut. The resident gut microbiota also serves as a major barrier that prevents the ectopic colonization by swallowed oral bacteria, due to its colonization resistance. Therefore, disruption of the healthy gut microbiota results in the increased colonization of the gut by oral bacteria. For instance, the antibiotic vancomycin is used to treat bacterial infections, and is known to perturb gut microbial composition, which generates niches for translocated oral bacteria to colonize and expand in the gut. For example, *Klebsiella* spp. are prominent in the saliva of patients with inflammatory bowel disease and are resistant to multiple antibiotics, including ampicillin (129). Ampicillin treatment can therefore result in the gut being colonized by oral *Klebsiella* spp. and a subsequent pathogenic Th1 cell expansion in the gut. Therefore, inadequate antibiotic use and other gut dysbiosis–inducing factors may increase the opportunistic gut colonization by oral bacteria (130). As mentioned, gastric acidity is another important hindrance for oral bacteria to reach the gut. Patients who suffer from gastric dysfunction related to achlorhydria, due to long-term use of proton pump inhibitors, exhibit a significant increase in gut colonization by oral bacteria such as *Haemophilus* spp., *Streptococcus* spp. and *Veillonella* spp. Gastritis and gastric surgery may also result in reduced exposure of ingested oral bacteria to gastric juice (131, 132) and research has shown that individuals with gastritis or who had gastric surgery have an altered gut microbial composition, with a significant increase in the relative abundances of specific oral microbiome taxa, such as *Streptococcus* spp., *Veillonella* spp., and *Enterobacteriaceae*, in the gut. Moreover, certain oral bacteria, such as *P. gingivalis*, are acid tolerant and can consequently pass through the stomach barrier to the gut (133) (Figure 1).

Initial reports found that the taxonomic compositions of the gut and oral microbiotas were distinct; however, the community types at these two sites were predictive of each other (134). A more recent study investigated saliva and fecal microbial strain populations in 470 individuals from five countries and they reported common and extensive transmission to and colonization of oral microbes in the large intestine in healthy individuals. The vast majority of oral species were found to be transferable to the gut, with increased transmission levels reported for opportunistic pathogens and in patients with colorectal cancer and rheumatoid arthritis. It is therefore possible that the oral cavity may serve as an endogenous reservoir for gut microbial strains and the study by Ding and Schloss (134) provides evidence that oral-gut transmission is an important process that influences the gut microbial composition and could therefore indirectly influence CNS functioning by altering the gut microbiota. Furthermore, the oral-gut axis has the propensity to fuel systemic inflammation within a positive feedback loop. Not only does a dysbiotic oral microbiota directly fuel inflammation (*via* several mechanisms discussed earlier in the review), it can also alter the composition, functioning and microbial metabolites of the gut microbiota, which subsequently also results in a pro-inflammatory cascade, which further exacerbates inflammation in the oral cavity and diminishes oral health (Figure 1). The oral microbiota also has the potential to affect the gut microbiota *via* the release of metabolites and bacteriocins. In their natural environment, bacteria produce bacteriocins, peptides with antibacterial activity, in order to compete against other bacteria for nutrients (135). Even if there are no data currently available about the specific effect of oral microbial metabolites and bacteriocins on the composition of the gut microbiota, *in vitro* studies have shown how different commensal oral species, such as *Streptococcus sanguinis* or *Streptococcus oralis*, have antimicrobial properties *via* the release of bacteriocins under different experimental conditions. These effects may also occur in the gut (136); however, their *via*bility during transport and uptake and stability in the gastrointestinal tract are yet to be investigated.

The gastrointestinal tract consists of several parts, each with distinct microbial counts [colony forming units per milliliter (cfu/ml)] and compositions. The stomach has the lowest abundance of bacteria (1–10^2^ cfu/ml); the small intestine, the main digestion and absorption site, consists of the duodenum (10^1^-10^3^ cfu/ml), jejunum (10^3^-10^4^ cfu/ml) and ileum (10^7^-10^9^ cfu/ml), with increased bacterial abundance as we get closer to the colon, which has the highest bacterial load (10^11^-10^12^ cfu/ml). The majority of gut microbiome studies use stool samples, which is a good proxy for the colonic microbiome. However, the colonic microbiome differs significantly from the microbiome of the small intestine (137), which is located much closer to the oral cavity and might be the main site where oral microbes could render an effect. The small intestine is the main site for immune surveillance in the gut (138, 139) and has more than 100 times the surface area compared with the large intestine. Its mucus layer is also thinner and more permeable, potentially providing closer interactions between its microbes and the host (140). A recent study found a clear relationship between the oral microbiota and duodenal microbiota. In addition, they reported an association between the absolute abundance of disruptor taxa (taxa that displace the strict anaerobic taxa common in the duodenum, including *Klebsiella, Escherichia, Enterococcus*, and *Clostridium*), small intestinal bacterial overgrowth (SIBO), and the prevalence of severe gastrointestinal symptoms (141). It is therefore plausible that the strongest effects of the oral microbiome on the gut may occur in the small intestine and future studies should investigate this in more detail, in conjunction with immune and tight junction marker measurements.

### How the Periodontal Microbiota Influences the CNS and Behavior

Periodontal bacteria and bacterial molecules have several possible means whereby they can impact the CNS. They can directly invade the brain *via* the bloodstream or *via* cranial nerves (142). Lipopolysaccharides can lead to the deterioration of the blood–brain barrier (BBB) and increase its permeability, thereby providing circulating periodontal bacteria/bacterial molecules the opportunity to penetrate into the CNS through a compromised BBB (21, 143). Other points of entry include the circumventricular organs and choroid plexus (which lack a contiguous BBB) (144), as well as the olfactory and trigeminal nerves (145). Periodontal bacteria and/or bacterial molecules can also communicate with brain-resident microglia through the leptomeninges (146). Furthermore, periodontitis induces systemic inflammation and pro-inflammatory cytokines can activate endothelial cells that express receptors for TNF-α and IL-1, which, in turn, signal to the perivascular macrophages located immediately adjacent to cerebral endothelial cells. These perivascular macrophages subsequently communicate with microglia and thus lead to microglial activation and subsequent neuroinflammation (147, 148). Another potential mechanism whereby the oral cavity could influence the immune system is *via* exosomes, a specific subgroup of extracellular vesicles that are secreted by cells and serve as important mediators of intercellular communication. These nanoscale lipid bilayer structures contain various molecules, such as proteins, lipids, miRNAs, mRNA, and many other non-coding RNAs. Exosomes are present in most body fluids, including saliva and one study found that reduced levels of CD9/CD81 exosomes in saliva were associated with the pathogenesis of the periodontal disease (149). Another study compared the salivary exosomal proteins in young adults with severe periodontitis and healthy individuals (using mass spectrometry and gene ontology analysis) and identified 26 immune-related proteins that were unique to severe periodontitis (150). Periodontal bacteria therefore have several mechanisms whereby they can transduce peripheral inflammation, including periodontitis, into neuroinflammation, and thereby influence CNS functioning and behavior (Figure 1).

### How Stress Influences the Oral Microbiota

Chronic psychological distress dampens diurnal salivary glucocorticoid and catecholamine secretion levels [as assessed by measurement of salivary cortisol (151) and alpha-amylase (152), respectively]. Glucocorticoids and catecholamines are able to modulate gut microbes (153, 154) and may therefore also blunt diurnal rhythms in relative abundance and/or functional microbial pathways within the oral cavity (155). Indeed, research has shown that for the salivary microbiota, considerable diurnal variability exists within and between individuals (156), and that these levels differ between highly stressed individuals compared to those with lower stress (as previously discussed in this manuscript) (102). The relative abundances of several taxa within the oral microbiota have also been associated with anxiety and stress-related phenotypes; however, these are all cross-sectional studies, and it is therefore uncertain which was the driving factor: stress or the oral microbiota. In this instance animal models are useful to try and identify the causal factor. In a pre-clinical study mentioned earlier, Martinez et al. (93) induced periodontitis in rats, followed by CMS to induce a depression-like syndrome and elegantly illustrated that pre-existing periodontitis together with subsequent stress exposure resulted in worse periodontal outcomes, exacerbated inflammatory profiles and HPA axis dysregulation, compared to animals without periodontitis. There is a lack of pre-clinical and clinical studies that focus on this research question and future studies should identify individuals with pre-existing periodontitis and perform longitudinal follow-up studies to monitor mental health outcomes.

## The Oral Microbiota as a Potential Therapeutic Target

Considering the significant effect that periodontal health plays in systemic diseases, mainly *via* inflammatory cascades, it is imperative to maintain oral health, to protect the periodontium from pathogens that promote inflammation and result in periodontitis. Periodontal therapy in patients with periodontitis uses a stepwise approach based on supra- and sub-gingival debridement (either non-surgical or surgical) with the potential application of antiseptics and/or antimicrobials in specific cases to control biofilms and limit the presence of pathogenic bacteria (157). Good oral hygiene is the first line of defense and the primary preventative measure of oral diseases. In addition to daily oral hygiene routines, supra and subgingival instrumentation can also be performed by dental professionals to remove dental biofilm and calculus on tooth surfaces. In the most severe cases, surgical treatment, and a multidisciplinary phase (e.g., restorative treatment or orthodontic treatment) might be required. These procedures, in conjunction with daily hygiene practices, can reduce tissue inflammation and PPDs (157), improving or maintaining clinical attachment levels. It is important to note that, in the short-term, exacerbation of systemic inflammation is expected following dental procedures in some specific patients; however, in the long-term, improvement of systemic inflammatory biomarkers becomes more evident (158, 159).

Although no research is currently available that shows how targeting the periodontal microbiota could improve mental health outcomes, it's clear that treatment of periodontal diseases promotes oral health (160, 161) by lowering the levels of inflammation and *via* several indirect pathways between the oral and gut microbiotas and the CNS (as discussed earlier in the manuscript), these interventions may also have the potential to influence mental health outcomes.

One study investigated the effects of the anti-inflammatory agent, aspirin, on pro-inflammatory taxa of the gut and oral microbiota, to determine whether the well-known protective effect of aspirin for colorectal cancer risk, is mediated *via* the microbiota. Firstly, they performed a randomized placebo-controlled trial in healthy individuals to study the effect of a 6-week aspirin intervention on the relative abundance of pro-inflammatory oral taxa. Aspirin treatment altered the relative abundance of 9 of the 12 pre-specified pro-inflammatory taxa (at genus level), suggesting that aspirin may alter the relative abundance of taxa associated with oral dysbiosis and inflammation. Secondly, they evaluated the effect of this 6-week aspirin treatment on inferred functional traits linked to LPS—a key bacterial metabolite with inflammatory properties. They did not find an association between aspirin treatment and the change in relative abundance of inferred functional traits for LPS. They did however observe a positive correlation between the relative abundance of inferred functional traits of LPS and the relative abundance of oral taxa associated with colorectal cancer risk and inflammation. Lastly, they performed a double-blind placebo-controlled trial to investigate whether pro-inflammatory oral and gut taxa are correlated and whether oral and gut microbial communities respond to an anti-inflammatory agent in a similar way. They found that aspirin induced changes in the alpha diversity of oral and gut microbiotas in a similar fashion. They also noted an inverse correlation between gut taxa that produce short-chain fatty acids (SCFAs) and pro-inflammatory oral taxa, therefore illustrating how particular taxa in the gut and oral microbiota may influence each other and the functions they perform (162).

A randomized placebo-controlled clinical trial evaluated the effects of probiotic lozenges containing *Lactobacillus reuteri* DSM 17938 (*L. reuteri* DSM 17938), as an adjunct to SRP to treat periodontitis. Periodontitis patients (*n* = 30) were monitored clinically and microbiologically at baseline, 3, 6, 9, and 12 weeks after therapy. Treatment consisted of a one-stage full-mouth disinfection (all patients), followed by sub-group assignment to either SRP + probiotic lozenges (*n* = 15) or SRP + placebo lozenges (*n* = 15) groups, where lozenges were used twice a day for 12 weeks. Their results showed a significant reduction in all clinical parameters for both groups after 12 weeks. Significant PPD reduction and CAL gain was evident in moderate and deep pockets and a significant reduction in *P. gingivalis* was observed in the SRP + probiotic group. The authors therefore concluded that oral administration of *L. reuteri* DSM 17938 could be a useful adjunct treatment to SRP in the management of periodontitis (163).

Several studies have investigated whether probiotics could improve oral health (as reviewed by Nguyen et al., 2021), and these have yielded mixed results (160). A recent meta-analysis was conducted to determine the effect of probiotics on gingival inflammation and the oral microbiota. The study included eleven randomized controlled trials and a total of 554 patients. They found oral probiotics had no significant improvement in the PI, GI and BOP of patients with dental biofilm-induced gingivitis. Furthermore, there were no significant differences in the volumes of GCF, the concentration of IL-1β, and the counts of *P. gingivalis, A. actinomycetemcomitans, F. nucleatum*, and *P. intermedia* between the group treated with probiotics and the placebo group. They concluded that there is no clear evidence that oral probiotics elicit positive effects on gingival inflammation and the oral microecological environment in patients with dental biofilm-induced gingivitis (161).

A recent randomized, double-blind, placebo-controlled study examined the adjunctive effect of a *L. reuteri* probiotic (*L. reuteri* ATCC PTA 5289 and *L. reuteri* DSM 17938) on the re-instrumentation of residual pockets in periodontitis patients. The study included 39 previously non-surgically treated periodontitis patients. Re-instrumentation was carried out, followed by application of probiotic and/or placebo drops. Patients received probiotic lozenges to use twice a day for 12 weeks. The results indicated that probiotic drops had no significant clinical effect. However, probiotic lozenges significantly decreased the overall PPD [2.64; standard deviation (SD) = 0.33 mm] after 24 weeks compared to the control lozenges (2.92; SD = 0.42 mm). This difference was even more noticeable in moderate and deep pockets. Authors also reported that significantly more pockets converted from ≥4 mm baseline measure to ≤ 3 mm at 24 weeks in the probiotic lozenges group and that there were fewer sites in need of surgery. However, despite the effects of the probiotic lozenges on PPD and pocket depths, no differences in microbiological composition of periodontopathogens were noted following probiotic use (164).

Several commercial products have been developed that specifically target the oral microbiota to promote oral as well as general health including a formulation containing *L. reuteri* DSM 17938 and *L. reuteri* PTA 5289 (165). Other products include an *adjunctive* oral microbiome therapy to help treat periodontal diseases, i.e., prebiotic toothpaste. However, only one the companies provide supporting scientific evidence to show the efficacy of their products (163, 164). Access to these products is unfortunately limited by the costs of the products and a general lack of awareness around oral health (166). Cost-effective oral health strategies to culture a healthy oral microbiota include good oral hygiene, low sugar, avoidance of tobacco use (167) and a cautious use of antibiotics (166). Since what happens in the mouth does not stay in the mouth, and a dysbiotic oral microbiota has been associated with several diseases, it is imperative that preventive strategies, including good oral hygiene practices and risk factor control, be taught from a young age, promoted at schools, tertiary education centers and community and health centers as an easy method to lower the risk of several diseases.

Future studies should include large scale, double blind, placebo controlled randomized clinical trials to determine the effects of periodontal microbiota-targeted interventions on mental health outcomes as a conjunctive treatment together with prescribed medications.

## Conclusions

There is an epidemiological association between diseases with altered oral microbiota such as periodontitis and anxiety, mood and trauma- and stress-related disorders, as reported by systematic reviews and meta-analyses. Less data are available for anxiety disorders and trauma-related disorders as compared to depression; however, the data suggest a role for the oral microbiota in these disorders. There is a need for more research in these disease areas, with in-depth, longitudinal, clinical characterization (periodontal, psychiatric, microbiota-related, and immunological) in large community samples to follow the progression of both periodontal diseases and neuropsychiatric disorders, to unravel the relationship between them and to identify molecular mechanisms involved in this relationship. Pre-clinical models enable researchers to investigate the intricate relationship between periodontitis and mental health outcomes, as one pathology can be induced prior to the other. For instance periodontitis can be induced and followed by subsequent models to induce anxiety- and/or depression-like endophenotypes, to indicate how pre-existing periodontitis affects subsequent anxiety- and depressive-like behaviors, or *vice versa*. These models also provide access to CNS and other tissues, which can be used to study immune profiles, LPS levels, bacterial translocation, markers of HPA axis dysregulation, and neuroinflammation. Since the periodontal microbiota can serve as a reservoir for the gut microbiota (especially in the context of an altered gut microbiota or in the case of acid-resistant taxa), more studies are needed that investigate both the oral and gut microbiotas in these neuropsychiatric disorders, to broaden our understanding of their interactions and bi-directional communication mechanisms to determine how they influence each other and mental health outcomes. This can be supplemented with functional intestinal organoid models (miniature organs grown *in vitro*) to evaluate the effect of oral pathogens on bacterial adherence, intestinal epithelial invasion, intestinal structural integrity and immune marker profiles. Similar to the gut microbiota, the oral microbiota is a tractable therapeutic target. More research is required to determine the clinical efficacy of the available products and to identify new targets. This could help maintain a healthy oral microbial community that is expected to contribute to improve general as well as mental health.

## Author Contributions

SM-M initiated and led this project. MM, TP, BG-B, EF, CL, JL, and SM-M contributed to the individual sections of this review. MM and SM-M merged the entire review. All the authors discussed and reviewed the manuscript.

## Funding

This study is supported by Santander-University Complutense of Madrid (PR41/17-20979) (EF), MINECO-FEDER (PD2019-109033RB-100) (JL and EF), SAF2017-85888-R (BG-B), and CIBERSAM-ISCIII (JL). SM-M is supported by a Brain and Behaviour Research Foundation NARSAD Young Investigator Grant (27050) and UNA4CAREER H2020-Marie Skłodowska-Curie Actions co-fund research grant (UNA Europa, an alliance of Universities FOR the Emergence of Talent and the Development of Research CAREERs; Grant number 847635). TP's work on this project was supported by the Rocky Mountain MIRECC, Aurora Colorado and VISN 5 MIRECC, Baltimore, Maryland, Veterans Health Administration.

## Author Disclaimer

This views expressed in this review belong to the authors and cannot be construed as representing the official views of NARSAD, Veterans Health Administration or other funders.

## Conflict of Interest

CL serves on the Scientific Advisory Board of Immodulon Therapeutics, Ltd., and is Cofounder and Chief Scientific Officer of Mycobacteria Therapeutics Corporation. EF reports contracts through University with Dentaid and Lacer, and personal fees for lecturing from Oral-B and Colgate. The remaining authors declare that the research was conducted in the absence of any commercial or financial relationships that could be construed as a potential conflict of interest.

## Publisher's Note

All claims expressed in this article are solely those of the authors and do not necessarily represent those of their affiliated organizations, or those of the publisher, the editors and the reviewers. Any product that may be evaluated in this article, or claim that may be made by its manufacturer, is not guaranteed or endorsed by the publisher.

## Abbreviations

BBB: blood-brain barrier
BDNF: brain-derived neurotrophic factor
BOP: bleeding on probing
CAL: clinical attachment level
CI: confidence interval
CMS: chronic mild stress
CNS: central nervous system
CPI: community periodontal index
CRP: C-reactive protein
EPM: elevated plus-maze
GCF: gingival crevicular fluid
GI: gingival index
HPA: hypothalamic-pituitary-adrenal
HR: hazard ratio
IL: interleukin
LPS: lipopolysaccharide
MAMP: microbe-associated molecular pattern
NF-kB: nuclear factor kappa-light-chain-enhancer of activated B cells
NPD: neuropsychiatric disorder
OFT: open-field test
OR: odds ratio
PAMP: pathogen-associated molecular pattern
PI: plaque index
PPD: probing pocket depth
PTSD: post-traumatic stress disorder
SCFA: short-chain fatty acid
SD: standard deviation
SRP: scaling and root planing
Tfh: follicular helper T cells
Th1: T helper 1
Th2: T helper 2
Th17: T helper 17
TLR: toll-like receptor
TNF: tumor necrosis factor
Treg: regulatory T cells
WHO: World Health Organization.
